# 电刀切割和机械切割在全胸腔镜肺段切除术段间平面分离中应用的对照研究

**DOI:** 10.3779/j.issn.1009-3419.2017.01.06

**Published:** 2017-01-20

**Authors:** 海波 刘, 钢 林, 诗杰 张, 伟明 黄, 学谦 商, 简 李

**Affiliations:** 100034 北京，北京大学第一医院胸外科 Department of Thoracic Surgery, Peking University First Hospital, Beijing 100034, China

**Keywords:** 胸腔镜, 肺段切除术, 切割缝合器, 电刀切割, 段间平面, Video-assisted thoracic surgery, Segmentectomy, Stapling device, Elcetrocautery, Intersegmental plane

## Abstract

**背景与目的:**

全胸腔镜肺段切除术随早期肺癌的高检出率逐渐受到关注，其中肺段切除术段间平面分离最常用的方法是电刀切割手工缝合和应用直线切割缝合器机械切割两种。但仅有很少的研究对两者进行对比，且均针对开放式肺段切除术，目前尚未有相应的研究针对全胸腔镜肺段切除术。本研究旨在探讨两种方法在全胸腔镜手术中的应用及安全性对比。

**方法:**

回顾性分析2013年9月-2016年3月北京大学第一医院胸外科行全胸腔镜肺段切除术的连续58例患者，根据段间平面分离方法不同分为电刀切割组30例和机械切割组28例，对两组患者手术时间、出血量、术后胸管留置时间、术后住院时间、胸腔引流量及术后并发症进行比较。

**结果:**

除手术时间[电刀切割组（248.70±54.46）min和机械切割组（209.39±67.25）min]两组间有统计学差异（*P*=0.017）外，术中出血量（60.00 mL *vs* 65.00 mL）、胸腔引流总量（445.00 mL *vs* 590.00 mL）、术后3天胸腔引流量[（455.33±318.333）mL *vs*（422.32±194.95）mL]、术后胸管留置时间（3.50天*vs* 4.00天）和术后住院时间（6.00天*vs* 6.00天）、术后并发症发生率（5/30 *vs* 2/28），两组差异均无统计学意义。

**结论:**

全胸腔镜肺段切除术段间平面的分离方法中，应用电刀切割手工缝合手术时间相对较长，但安全性不劣于应用切割缝合器机械切割缝合。

## Abstract (en)

**Background and objective:**

Complete thoracoscopic segmentectomy gained great attention with the high detection rate of early lung cancer. Electrocautery and stapler are most commonly used in dividing the intersegmental plane in pulmonary segmentectomy. However, few reports comparing the two methods exist; all of which contrapose an open approach because complete thoracoscopic approach is not mentioned. The aim of this study is to evaluate and compare the safety and efficacy of the two methods in intersegmental plane dissection during complete thoracoscopic pulmonary segmentectomy.

**Methods:**

A retrospective review of prospectively collected data was obtained for 58 consecutive patients who were treated by segmentectomy between September 2013 and March 2016 at a single center. The patients were divided into two groups according to the methods in intersegmental plane dissection. Thirty patients underwent intersegmental plane dissection with electrocautery (electrocautery group), and 28 patients underwent the same process using staplers (stapler group). Preoperative, intraoperative, and postoperative variables for patients were compared between two groups.

**Results:**

The operative time of electrocautery group was longer than that of the stapler group [(248.70±54.46) min *vs* (209.39±67.25) min, *P*=0.017]. Furthermore, no statistical difference was found between two groups in intraoperative blood loss (60.00 mL *vs* 65.00 mL), total drainage volume (445.00 mL *vs* 590.00 mL), drainage volume in first 3 days after surgery [(455.33±318.333) mL *vs* (422.32±194.95) mL], duration of chest tube drainage [(4.20±2.07) d *vs* (4.11±1.61) d], postoperative hospital stay [(6.33±2.98) d *vs* (5.89±1.55) d], and incidence of minor postoperative complication [16.7% (5/30) *vs* 7.1% (2/28)].

**Conclusion:**

Although operative time was longer in electrocautery group than in stapler group, using electrocautery for intersegmental plane dissection in complete thoracoscopic segmentectomy appeared to be a very safe and feasible procedure compared with stapler.

由于健康体检的普及，越来越多的微小肺癌被早期发现^[1, 2]^；虽然尚无多中心随机对照的大规模临床研究证实，但肺段切除术在早期肺癌外科治疗中的作用正逐渐得到重视。随着微创手术技术的迅速发展，全胸腔镜肺段切除术也受到更多胸外科医生和患者的青睐。肺段切除术的手术技术要求较高，其中段间平面分离肺实质切除最常用的方法是电刀切割手工缝合（electrocautery handsewing）和应用直线切割缝合器机械切割（mechanical stapling）两种。但仅有很少的研究对两者进行对比^[3, 4]^，且均针对开放式肺段切除术，目前尚未有相应的研究针对全胸腔镜肺段切除术。因此，我们对2013年9月-2016年3月进行全胸腔镜肺段切除术的58例患者进行回顾性分析，对两种段间平面分离方法进行对比。

## 1 研究对象和方法

### 1.1 研究对象

2013年9月-2016年3月间北京大学第一医院胸外科共96位例患者行肺段切除术，其中胸腔镜辅助小切口肺段切除术患者38例（其中包括2例因术中肺动脉损伤由全胸腔镜操作中转为胸腔镜辅助小切口手术），其余全胸腔镜肺段切除术的患者共58例纳入本研究。术前均行血尿常规、肝肾功能、凝血功能、血气分析、肺功能、心电图、胸部增强计算机断层扫描（computed tompgraphy, CT）等常规检查；考虑恶性肿瘤的患者行头颅磁共振成像（magnetic resonance imaging, MRI）、腹部超声或CT、骨扫描或全身正电子发射型计算机断层显像（positron emission tomography/computed tompgraphy, PET/CT）等排除远处转移。

纳入标准：①周围型病灶为转移癌或良性病变或癌前病变，行单纯肺楔形切除较困难；②早期周围型肺癌，直径≤2 cm，且CT显示结节内毛玻璃样成分≥50%，或影像学检查随诊证实肿瘤倍增时间较长（≥400天），或术中冰冻病理证实为微小浸润癌，且术中冰冻病理证实纵隔淋巴结无转移；③多原发肺癌，行分期或同期肺切除，难以耐受肺叶切除；④早期周围型肺癌，心肺功能较差或基础合并症较严重，难以耐受肺叶切除。排除标准：①未行完全胸腔镜肺段切除术；②同期双侧肺段切除。

根据肺段间平面的分离方法不同分为电刀切割（electrocautery group）和机械切割（stapler group）两组。选择段间处理方式时主要依据术者对病灶部位和段间边界形态的判断，同时兼顾术者的习惯；如病灶距离段间切缘较远，且段间边界较规整平直，则主要选择机械切割；如果病灶距离段间切缘较近，为避免切缘阳性，或段间边界不规整，难以应用切割缝合器时，则主要使用电刀切割。

### 1.2 手术方法

采用全身麻醉，双腔支气管插管，单肺通气；如患者不能耐受持续单肺通气则间断双肺通气。所有手术经由3位主刀医生完成。第六肋间腋后线1.0 cm切口置入30度胸腔镜摄像头做观察孔，第四肋间腋前线行3 cm-5 cm切口放置切口保护套做操作切口，不撑开肋骨，行单操作孔完全胸腔镜手术。先解剖处理相应肺动脉和肺静脉分支（1号丝线近心端双重结扎或内镜用直线切割缝合器2.5 mm钉高钉仓切断），再游离阻断确认后处理段支气管（3-0可吸收线连续缝合或内镜用直线切割缝合器4.5 mm钉高钉仓切断），最后应用电刀电凝切割（电刀输出功率为45 w；肺残面应用3-0可吸收线连续缝合）或内镜用直线切割缝合器（4.8 mm钉高钉仓）切断分离段间平面。段间平面采取根据肺动脉和肺静脉走行的解剖性标志和段支气管处理后双肺通气沿萎陷与膨复的肺组织边界相结合的方式来确立。除原位癌外，所有肺癌患者同期行纵隔淋巴结清扫；左侧清扫第4、5、6、7、8、9、10、11、12组淋巴结；右侧行第2、3A、4、7、8、9、10、11、12组淋巴结。术后经观察孔放置28 F胸腔引流管1根。

### 1.3 术后处理

全组患者术后清醒拔管转入普通病房。术后1天-3天均使用术后患者连硬外自控镇痛。拔胸管指征：拔管前24 h胸腔引流＜250 mL，肺复张良好并且无漏气。

### 1.4 观察指标

记录两组患者手术方式、手术时间、术中出血量、胸管留置时间、术后住院时间、胸腔引流量、术后并发症等情况。

### 1.5 统计学方法

选用SPSS 22.0统计软件进行分析，计量资料以均数±标准差（Mean±SD）或中位数表示，组间比较采用*t*检验或*Mann*-*Whitney U*检验；计数资料比较采用χ^2^检验或*Fisher’s* 精确检验；以*P*＜0.05为差异有统计学意义。

## 2 结果

全组共58例患者，其中电刀切割组30例，机械切割组28例。两组患者在性别、年龄、体重指数、吸烟史和术前肺功能状况、合并症、病理学特征等临床特征之间无明显差异（表 1）。切除的肺段解剖分布如表 2所示。

**1:** 电刀切割组和机械切割组患者临床病理资料 Clinicopathologic characteristics of the electrocautery and stapler groups

Characteristics	Electrocautery group (*n*=30)	Stapler group (*n*=28)	*P* value
Age (yr)	60.10±13.13	63.61±10.48	0.268
Gender			0.621
Male	12	13	
Female	18	15	
FEV1 (L)	2.43±0.57	2.46±0.84	0.877
FEV1 (%)	96.82±16.81	101.41±20.61	0.354
DLCO (%)	90.74±19.66	91.55±15.80	0.863
BMI			
Smoking history			0.835
Yes	6	5	
No	24	23	
Comorbidities			
Hypertension	11	12	0.630
Diabetes mellitus	5	2	0.425
Coronary artery disease	2	2	0.999
Arrythmia	2	3	0.665
Pulmonary artery hypertension	1	0	0.999
Stroke	0	1	0.483
COPD	7	8	0.649
Lobectomy history	2	5	0.246
Histology			
Single primary lung cancer	16	13	0.599
Metastatic tumor	1	5	0.097
Multiple primary lung cancers	5	5	0.905
Benign disease	6	2	0.256
Carcinoma *in situ*	2	3	0.665
FEV1: forced expiratory volume in one second; BMI: body mass index; COPD: chronic obstructive pulmonary disease; DCLO: diffusing capacity of the lung for carbon monoxide.			

**2:** 电刀切割组和机械切割组患者切除的肺段分布情况 Location of resected segments in the electrocautery and stapler groups

Segments	Electrocautery group (*n*=30)	Stapler group (*n*=28)
Right		
S2	3	1
S26	3	0
S3	1	0
S6	4	5
S7	1	0
S78910	0	1
S8	1	1
Left		
S12	0	1
S123	6	10
S2	1	1
S4	0	1
S45	3	3
S6	6	4
S8	1	0

全组患者无术中严重并发症和围术期死亡。术后病理证实段间切缘及支气管切缘均为阴性。除手术时间[电刀切割组（248.70±54.46）min，机械切割组（209.39±67.25）min]，两组间有统计学差异（*P*=0.017）外，术中出血量、是否行纵隔淋巴结清扫、术后拔管时间和住院时间等，两组差异均无统计学意义；全组有1例电刀切割组患者因胸腔粘连较重、创面渗血较多致术中出血量＞200 mL，其余患者术中出血量均≤200 mL（表 3）。

**3:** 电刀切割组和机械切割组患者的手术相关资料 Surgical data of the Electrocautery and Stapler groups

Characteristics	Electrocautery group (*n*=30)	Stapler group (*n*=28)	*P* value
Operative time (min)	248.70±54.46	209.39±67.25	0.017
Intraoerative blood loss (mL)	60.00	65.00	0.261*
Duration of chest tube drainage (d)	3.50	4.00	0.774*
Duration of hospital stay after surgery (d)	6.00	6.00	0.800*
Drainage volume in first 3 d after surgery (mL)	455.33±318.33	422.32±194.95	0.639
Total drainage Volume (mL)	445.00	590.00	0.663*
Intraoperative blood loss more than 200 mL	1	0	0.999
Mediastinal lymphonectomy			0.096
Yes	15	20	
No	15	8	
* *Mann*-*Whitney* *U*-test.			

全组共7例患者出现术后并发症（表 4）。有3例患者胸腔闭式引流超过7天，其中1例机械切割组患者因术后引流量较多第8天拔除引流第9天出院；1例肺不张的电刀切割组患者采用气管镜吸痰并重新放置胸管后于术后第9天拔除引流，第13天好转出院；全组只有1例电刀切割组患者肺漏气超过7天，留置胸腔引流11天后肺漏气停止，肺复张良好，拔除胸管，术后13天出院。1例乳糜胸的患者采用禁饮食静脉营养保守治疗后，于术后第7天拔除引流好转出院。1例肺部感染的患者应用抗生素、支气管扩张剂等药物治疗后于术后第6天拔除引流第15天好转出院。1例房颤的患者予以应用可达龙后转复。1例声带麻痹考虑喉返神经损伤的患者未行特殊处理。两组间无论单项或总并发症发生率均无统计学差异（*P*=0.425）。

**4:** 电刀切割组和机械切割组患者的术后并发症资料 Postoperative complications of the Electrocautery and Stapler groups

Complications	Electrocautery group (*n*=30)	Stapler group (*n*=28)	*P* value
Atrial fibrillation	0	1	0.483
Atelectasis	1	0	0.999
Chylothorax	1	0	0.999
Pneumonia	1	0	0.999
Vocal cord paralysis	1	0	0.999
Prolonged air leak (＞7 d)	1	0	0.999
Prolonged chest tube drainage (＞7 d)			0.999
Atelectasis	1	0	
Air leak	1	0	
More drainage volume	0	1	
Total morbidity	5*	2	0.425
*Two patients had two complications.			

## 3 讨论

由于健康体检的普及，尤其是对低剂量CT在高危人群筛查中获益的肯定^[5]^，肺癌治疗的疾病谱正在发生变化，越来越多的微小肺癌被早期发现。尽管肺叶切除加纵隔淋巴结清扫术仍然是肺癌手术治疗的金标准^[6]^，越来越多的证据^[7-9]^显示对于早期发现的微小肺癌肺段切除术可以达到相同的根治效果。由于保留了更多的肺组织，接受肺段切除术的患者术后肺功能储备更好，生活质量更高^[10-12]^。胸腔镜微创手术比传统开胸手术创伤更小，很多研究也已证实胸腔镜肺段切除术不仅可以达到开放肺段切除术一样的根治效果和安全性，而且可以更加微创，术后恢复更快，并发症发生更少^[13-15]^。因此应用胸腔镜肺段切除治疗符合适应征的早期微小肺癌已经成为一种趋势，无论对外科医生还是患者都存在着巨大的吸引力。

相比肺叶切除，肺段切除对术者的基本手术操作技巧和对肺脏解剖的掌握要求较高。段间平面的分离与闭合是手术中至关重要的环节，目前最常用的是电刀分离手工缝合和切割缝合器机械切割缝合两种方式。但只有很少的研究^[3, 4]^对两种段间平面分离方法进行对比，且均针对开放式肺段切除术。Miyasaka等^[3]^和Ohtsuka等^[4]^分别研究发现在开放式肺段切除术中，无论是手术时间还是手术安全性电刀切割与机械切割间均无明显差异。

应用切割缝合器进行机械切割时，在对段间平面进行分离的同时完成肺残面的闭合，比较方便快捷，可以节约手术时间；缝合钉闭合细密确切，也减少了术者对术中出血和术后肺残面漏气的顾虑。所以大部分医生倾向于采用切割缝合器进行对段间平面的分离与闭合^[16]^。但目前绝大部分切割缝合器钉仓尚不能随意弯曲，而段间平面多不平直，经常需要多切除一部分正常组织才能保证达到足够的安全距离，远没有电刀电凝分离时那么“适形”^[17]^。而切割缝合器钉仓闭合多过于紧密，有动物实验^[18]^显示可以导致保留的肺组织过于皱缩，复张受限。另外，应用切割缝合器分离段间平面更容易损伤段间静脉，导致保留的肺组织静脉回流受限^[18, 19]^。所以电刀切割手工缝合可能会更好地保留患者的肺功能。而且切割缝合器费用高昂，中国仍然是一个发展中国家，患者经济压力普遍较重，应用电凝分离替代机械分离无疑可以减少较大的医疗开支^[4, 20]^。此外，有人认为目前的直线切割缝合器钉仓仍较粗大，切割时切割刀对肺组织及肿瘤可能有一个挤压牵拉作用，导致实际的切缘比要求的安全切缘要小，更易导致切缘肿瘤细胞残留；而应用电刀切割则能有效避免上述缺点^[21]^。

完全胸腔镜下肺段切除术，技术更加复杂，操作更加精细，手术的难度更大；尤其因为切口位置大小和胸腔内器械角度的限制，对术者而言，段间平面的分离不啻为一个巨大的挑战。目前尚未发现有研究对全胸腔镜下两种段间平面分离方法进行对比。因此我们总结了我中心连续58例行全胸腔镜肺段切除患者的资料，发现无论术中出血、术后胸腔引流量还是术后拔管时间、术后住院时间，应用切割缝合器机械切割与应用电刀电凝切割两组患者均无明显差异，都可以达到满意的结果。因为腔镜下观察更加清晰，只要肺段血管辨别和分离无误，电刀切割段间平面过程中只要先闭合段间的小血管，肺实质的分离并不会导致出血太多^[3, 4]^。而肺段切除的肺组织容量较小，只要胸腔引流通畅和患者能有效咳痰，残留肺均可以满意的复张，术后不会留有明显的胸腔残腔。因此如果患者肺组织没有严重气肿，只要术中仔细结扎了段间平面明显的支气管断端，即使手工缝合段间平面时较为疏松，术后残面也会很快愈合，不会出现严重的漏气^[3, 4]^。所以我们全组患者均采用单纯缝线或机械闭合，未使用任何生物胶等辅助，除1例患者出现术后漏气超过7天外，其余患者均愈合良好。两组术后并发症发生率亦无统计学差异。因此，在手术安全性方面，应用电刀电凝切割段间平面比较应用切割缝合器机械切割没有明显的劣势。

我们在临床工作中发现，因为胸腔镜手术切口较小，切割缝合器伸入胸腔时存在角度限制，钉仓较粗大不能随意弯曲，有时进行段间平面分离比较困难；应用电凝分离时，长电刀柄较细，反而可以更容易地抵近电凝目标进行分离；而目前微创技术及器械的进步，使得有经验的胸外科医生进行腔镜下肺残面的缝合迅速确切，毫不费力。我们的研究发现电凝切割组比机械切割组手术时间要长，有统计学差异，主要原因在于电凝切割组中我们纳入了更多的段间分界不平直的病例，其手术操作更复杂，耗时更长；另外我们手术例数较少，早期病例尚处于学习曲线当中，腔镜下缝合技术还不够熟练也是很重要的因素^[22]^。

我们研究的不足之处在于病例数较少，且为回顾性研究；而且没有对术后肺功能和住院费用进行评估和统计，没有反映出电刀切割方法的优势。我们准备在以后的研究中进行改进。

总之，我们认为全胸腔镜肺段切除术段间平面的分离中，虽然应用电刀切割手工缝合手术时间相比较长，但安全性不劣于应用切割缝合器机械切割缝合。
